# Real-Time, Objective Assessment of Facial Paralysis Using a Mobile Tool (FaceADE): Feasibility Case-Control Study

**DOI:** 10.2196/85965

**Published:** 2026-07-14

**Authors:** Marcelina Puc, Katherine Guo, Anthony Newman, Alexandra Antoinette Myers, Amar Sheth, Jon-Paul Pepper

**Affiliations:** 1 Division of Facial Plastic and Reconstructive Surgery Department of Otolaryngology-Head and Neck Surgery Stanford Medicine Palo Alto, CA United States; 2 Thomas F. Frist, Jr. College of Medicine Belmont University Nashville, TN United States; 3 University of California Davis School of Medicine University of California, Davis Sacramento, CA United States

**Keywords:** facial paralysis, facial palsy, TrueDepth, clinical evaluation, recovery tracking, computer vision

## Abstract

**Background:**

Patients with facial paralysis require detailed clinical assessment and long-term follow-up to monitor facial function. The current standard of care for evaluating facial symmetry and movement uses validated clinician scoring tools such as the House-Brackmann facial paralysis score or the Electronic Clinician-Graded Facial Function Scale (eFACE). Existing tools are difficult to use in normal clinic workflows and do not provide real-time facial movement tracking, representing an unmet need. Therefore, we developed FaceADE, a novel iOS app leveraging native 3D image acquisition capabilities on the iPhone to rapidly quantify facial movement in patients with facial paralysis.

**Objective:**

This study aimed to benchmark FaceADE against 2D image analysis and establish the feasibility of measuring oral commissure movement in patients with facial paralysis and healthy controls.

**Methods:**

Patients were enrolled in a tertiary care clinic focused on facial paralysis treatment. Patients underwent image capture using the FaceADE app assisted by study team personnel. Measurements of lip commissure position and movement were obtained from 20 patients with facial paralysis and 10 healthy volunteers without facial paralysis. Measurements of lip movement and symmetry gathered from FaceADE were benchmarked against 2D measurements using open-source image analysis software (ImageJ).

**Results:**

FaceADE measurements of lip commissure position and movement showed strong agreement with 2D measurements in both healthy volunteer and facial paralysis cohorts. The intraclass correlation coefficient was 0.96 (95% CI 0.90-0.98; *P*<.001) in the healthy volunteer cohort and 0.82 (95% CI 0.72-0.88; *P*<.001) in the facial paralysis cohort. Bland-Altman analysis found strong agreement between the 2 methods for these measurements. The 95% CI contained 97.5% (39/40) of data points in the healthy cohort and 91.2% (73/80) of data points in the facial paralysis cohort.

**Conclusions:**

Our proposed mobile method of measuring clinically important lip commissure position and movement is feasible for use at the time of care delivery. In the future, this technology may be useful for a quantitative assessment of facial paralysis severity.

## Introduction

Facial paralysis encompasses conditions that cause weakness or paralysis of the face. The incidence of facial paralysis in the United States was approximately 47.4 per 100,000 in 2022 [1,2]. Patients with facial paralysis report lower quality of life and severe restriction of emotional expression, eye closure, and communication [3-5]. Interventions may include medical treatment, physical therapy, and facial reanimation surgery for persistent and severe facial paralysis [6-9]. Currently, disease progression and recovery are tracked over time via patient-reported outcome measures and clinician-scored metrics based on physical examination [10].

Facial paralysis is assessed by clinicians using several available scales, such as the Sunnybrook Facial Grading System, the Electronic Clinician-Graded Facial Function Scale (eFACE), and the House-Brackmann scale [11]. However, these scales may have significant interrater variability, and more detailed scales such as the eFACE may be difficult to complete in a busy clinical environment [12,13]. Thus, an automated and accurate measure of facial movement and symmetry could help standardize assessment and save valuable time during a clinical encounter.

Initial efforts to automate facial analysis have been made [14-19]. Existing methods allow for facial movement analyses from photographs or stored videos post hoc but do not offer real-time assessment and data output during the clinical encounter. Furthermore, they often rely on assumed average values, such as an iris size of 11.77 mm, to output common distance measures of facial movement [18,20].

We propose that Apple’s TrueDepth technology can be leveraged to provide real-time measurements of facial movement by creating an individualized 3D facial map [21-23]. For face recognition, the TrueDepth camera uses infrared (IR) projections to create a 3D map of the face. Facial features and movements can be measured despite variations in head tilt and skin tone [24].

We developed an iOS app, FaceADE, to obtain immediate measurements of clinically important facial features using Apple’s TrueDepth camera. In this feasibility study, we demonstrate that this method can accurately measure oral commissure position and movement based on benchmark comparisons to 2D still-frame analysis in both healthy volunteers and patients with facial paralysis.

## Methods

### App Development

**Figure 1:**
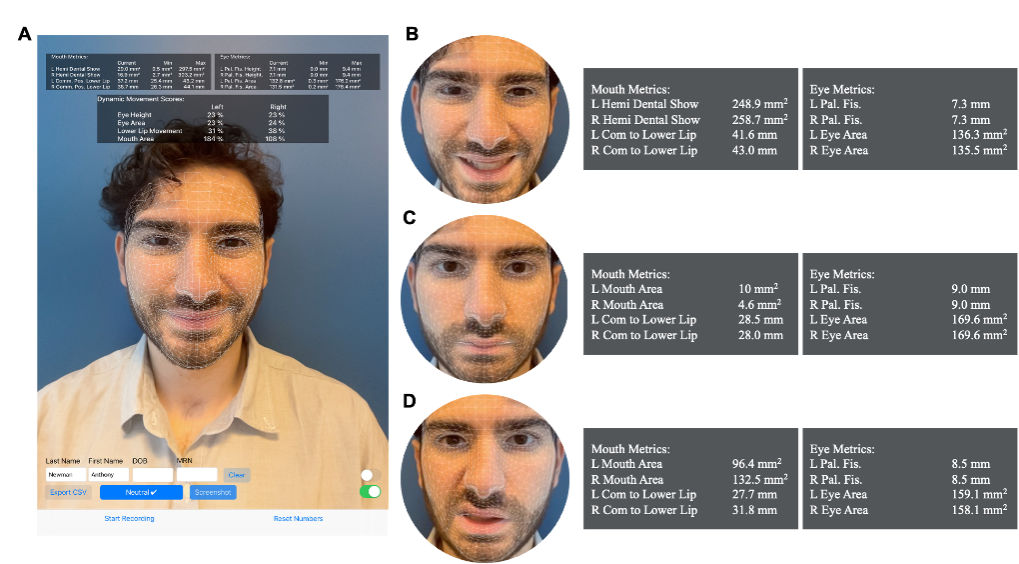
Sample FaceADE output data. (A) A screenshot of the FaceADE app with clinically important facial feature measurements. (B-D) Show sample data output for 3 different facial expressions.

FaceADE is an iOS app developed in Swift (Apple Inc) to analyze facial features and quantify clinically relevant physical measurements using Apple’s TrueDepth camera [25]. This camera tracks projected IR dots to create a real-time 3D digital mesh of the face that retains accuracy in variable light conditions [26,27]. Apple’s ARKit uses this system to create a 1220-point face mesh with the coordinate system based relative to the position of the head [28]. The FaceLandmarks tool was used to draw curvilinear lines between the predetermined vector points that designate specific facial features of interest on the left and right hemiface [26]. Measurements of these curves were then imputed in the 3D space and then displayed within the app (Figure 1). Facial measures include oral commissure position, mouth area, palpebral fissure height (eye height), and eye area. For this feasibility study, only oral commissure metrics were extracted and analyzed. The 16 facial measurements listed in Figure 1 were exported in a CSV file at 15 frames per second. Dynamic facial movement scores are reported as a percentage relative to the neutral expression, which can be set by the user at any point of the recording by pressing the “neutral expression” button. Patient demographic fields are shown in the lower left-hand corner above the “neutral expression” button. These fields allow for the labeling of patient data when they are exported after recording. In the lower right-hand corner, users can toggle on or off the face mesh, and to the right of the “neutral expression” button, there is a screenshot button to capture images while the video is recording. Measurements were captured at 15 frames per second on a tripod-stabilized iPad, and no video smoothing was applied. Measurements and vector coordinates of each frame of the video were collected to quantify movement during dynamic facial expressions. A walk-through video of FaceADE can be found in Multimedia Appendix 1. FaceADE is available for download on GitHub [29] and in the Apple App Store.

### Facial Feature Measurements

Although the entire matrix of the face was tracked and recorded, the facial feature emphasized in this feasibility study was the lower lip commissure position (mm), defined as the distance from the right or left oral commissure to the facial midline along the lower lip vermillion border (Multimedia Appendix 2). Oral commissure movement is one of the most clinically important features of facial movement and is the focus of many surgical procedures for facial paralysis [30].

### Ethical Considerations

Institutional review board approval was obtained (Stanford Institutional Review Board 79624), including an approved consent form and enrollment process. Patients aged 18 years and older of any gender were approved to be enrolled at the Stanford Facial Nerve Center. Eligible patients discussed study participation with a study team member in a private clinic room after their clinical encounter was complete. If they wished to participate in the study, patients subsequently completed the informed consent process. Study team members and clinical staff from the Facial Nerve Center served as controls. All patients and controls were assigned a sequential numerical identifier for data deidentification prior to analysis, and only study team members handled data for collection and analysis.

All images of individuals used in the manuscript are of study team members who provided explicit written consent for the use of their images and reviewed all figures prior to submission.

FaceADE videos were stored on encrypted and HIPAA (Health Insurance Portability and Accountability Act)-compliant iPads (iPad Pro; model number A2759; Apple Inc). Data were transferred from the iPads to a HIPAA-compliant server. Once videos were uploaded to the server, they were deleted from the iPads. All patient images were stored on the same secure server, and all data analysis was performed on a password-protected, encrypted, and HIPAA-compliant desktop computer.

### Data Collection

Patient videos were obtained at the conclusion of the clinical encounter in a private examination room. After obtaining patient consent, a ruler was used to make a 1-cm mark with a surgical marker on the patient’s forehead. Patients were seated on a stool, and the tripod with the iPad mounted was placed in front of them. Patients were asked to look directly into the camera during recording. Patients were read standardized face examination prompts (Multimedia Appendix 3) and asked to rest their face between each prompt. The video was then uploaded to a HIPAA-compliant server. Videos were used to obtain still frames of the patient’s neutral and full smile expressions selected from a representative video frame of the face after the patient received the face examination prompt from the recording (ie, “please give a big smile”). The still frames were opened in ImageJ and scaled using the 1-cm mark on the skin. The segmented line tool in ImageJ was then used to measure right and left commissure distance along the lower lip vermillion border. To avoid bias in ImageJ measurements and analysis, the videos had the face mesh turned off, and ImageJ data were entered into REDCap (Research Electronic Data Capture; Vanderbilt University) before FaceADE data were recorded. A study flowchart is shown in Multimedia Appendix 4.

### Analysis

To measure the overall agreement between 2D analysis and FaceADE, the measurements from both facial sides and expressions were pooled, and the intraclass correlation coefficient was calculated using a 2-way random-effects model where both patients and the 2 methods were considered to be randomly selected, and absolute agreement was used instead of the consistency of the trend. The reliability estimate of a single rating was generated. To estimate the potential bias between methods, we performed separate Bland-Altman analyses for each cohort. The bias was defined as the difference between every pair of FaceADE and ImageJ measurements. For each cohort, we used a linear mixed model to account for the measurement of both sides of the face from the same patient. The models included the bias as the outcome measure, facial side and expression as fixed effects, and patient as a random effect with random intercept. We calculated the total variance by adding the within- and between-subject variation generated from the model. The 95% limits of agreement were computed as the bias ±1.96√total variance. Standard Bland-Altman plots were created, with the bias plotted against the average of FaceADE and ImageJ measurements. A horizontal line was drawn at the mean difference and 95% limits of agreement. The scatter dots outside the 95% limits of agreement were identified as possible outliers. All analyses were conducted using RStudio (version 2026.01.2+418; Posit PBC).

## Results

Our healthy volunteer cohort was composed of 10 study team members, with 6 (60%) being female; the facial paralysis cohort included 20 patients, with 9 (45%) being female. The average age at paralysis onset was 45.5 (SD 21.1) years. The average duration of the videos was 78 (SD 27.5) seconds (Table 1).

**Table 1:** Characteristics of the participants enrolled in the study.

		Values		
		Facial paralysis (n=20)	Healthy volunteers (n=10)	
Age at facial paralysis onset (years), mean (SD)		45.5 (21.1)	—^a^	
Age at consent (years), mean (SD)		53.4 (16.5)	30.1 (7.8)	
**Sex, n (%)**				
	Female	9 (45)	6 (60)	
	Male	11 (55)	4 (40)	
**Race, n (%)**				
	Asian	7 (35)	4 (40)	
	White	7 (35)	5 (50)	
	Other	6 (30)	1 (10)	
**Ethnicity, n (%)**				
	Hispanic or Latino	6 (30)	2 (20)	
	Non-Hispanic or Latino	14 (70)	8 (80)	
**Reason for FP, n (%)**				
	Bell’s palsy	8 (40)	—	
	Ramsay Hunt syndrome	3 (15)	—	
	Iatrogenic	2 (10)	—	
	Malignancy	6 (30)	—	
	Right arteriovenous malformation	1 (5)	—	
**Laterality of facial injury, n (%)**				
	Left	9 (45)	—	
	Right	11 (55)	—	

^a^Not applicable.

**Figure 2:**
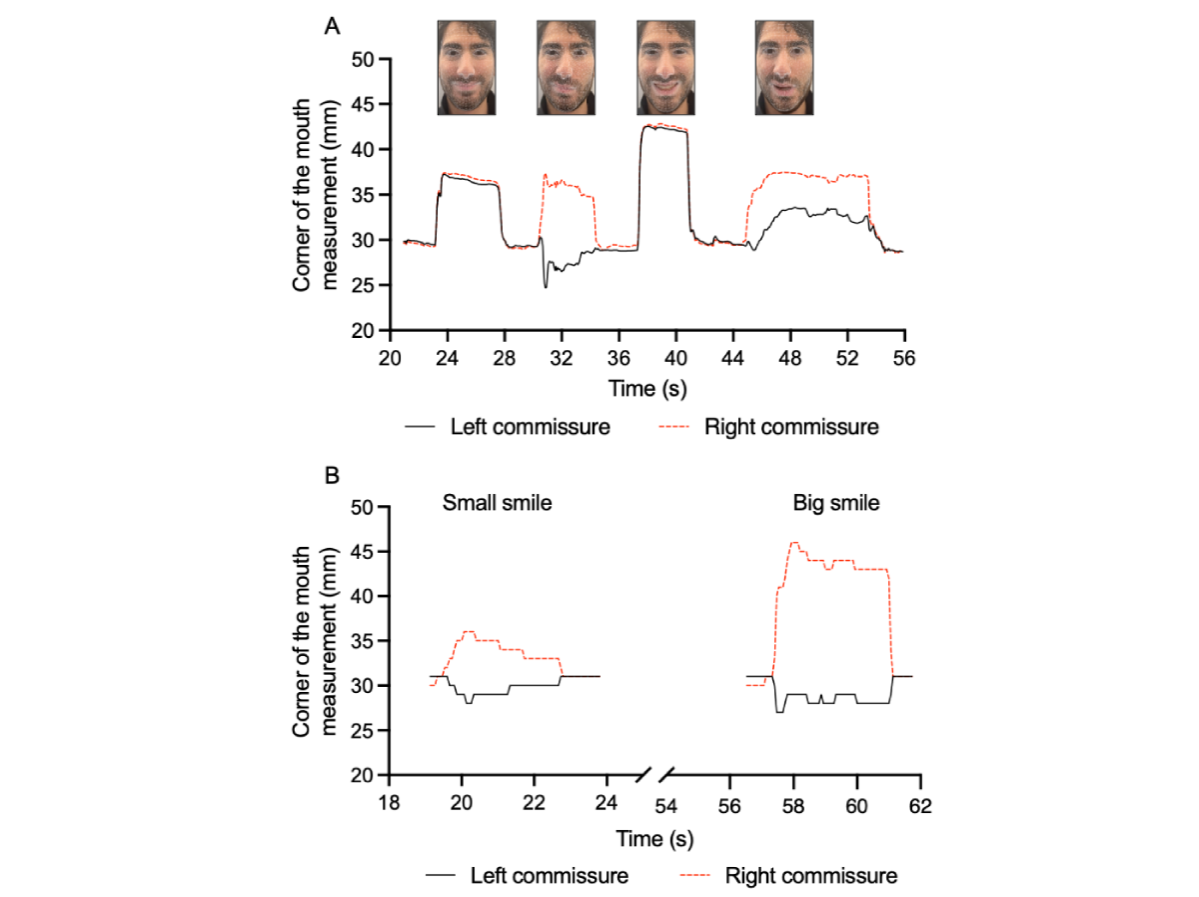
Corner of the mouth movement over time. (A) Lower lip commissure movement was tracked for a healthy volunteer, who performed small and big symmetrical smiles and simulated asymmetrical smiles. (B) Lower lip commissure movement in a patient with complete left-sided facial paralysis. Asymmetry of movement is observed, with larger asymmetry in the full smile than in the small smile. FaceADE is able to capture corresponding movements of the left commissure that are the result of unopposed movement on only one side of the face.

As a video- and IR-based method, FaceADE tracked facial movements over time. A healthy volunteer performed a small smile and a full smile with dental show, simulated a small asymmetrical smile, and simulated a larger asymmetrical smile with dental show (Figure 2A). In an asymmetrical smile, FaceADE revealed greater movement of the right commissure and tracked the distortion of the left commissure that resulted from unilateral movement on one side. Analysis of commissure movement during a smile in a patient with facial paralysis showed similar trends while tracking oral commissure movement throughout the facial expression (Figure 2B). A full smile with dental show resulted in greater commissure asymmetries than small smile expressions, and subtle changes in both commissures could be observed over the course of the movement.

**Figure 3:**
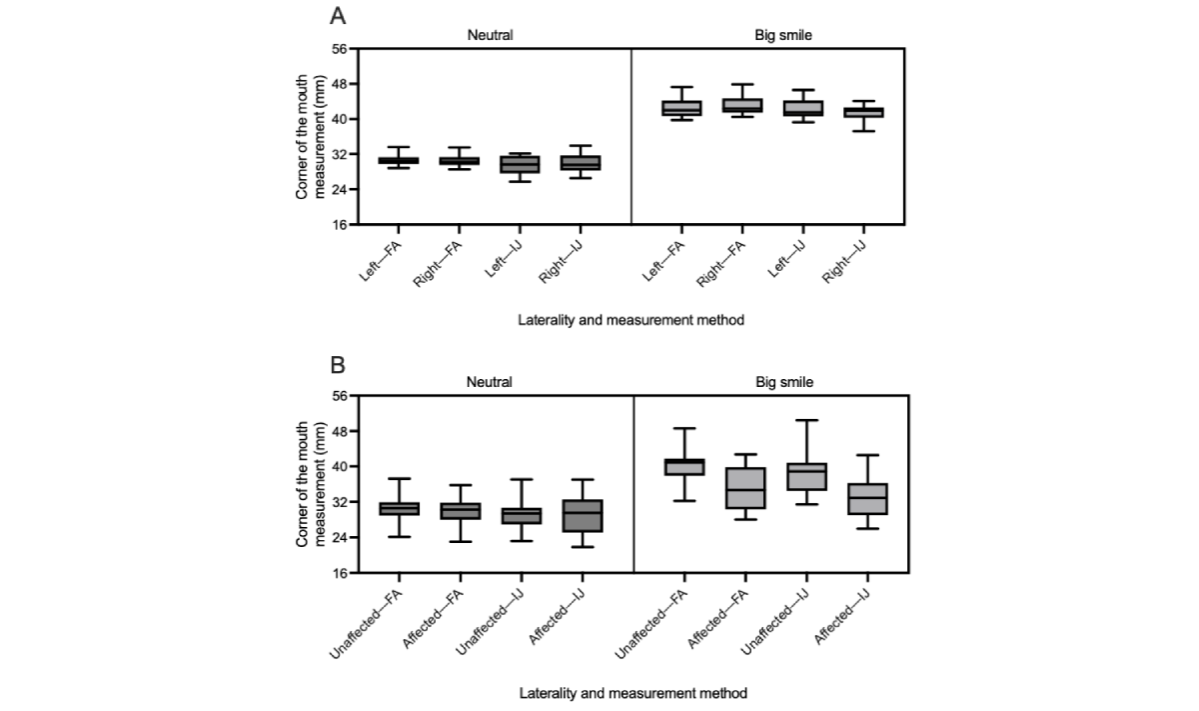
FaceADE (FA) measurements benchmarked against 2D measurements. (A) The distribution of commissure measurements in the healthy volunteer cohort (n=10) for both neutral and full smile expressions of each hemiface is displayed. (B) The distribution of commissure measurements in the facial paralysis cohort (n=20) for both neutral and full smile expressions of the affected and unaffected sides. IJ: ImageJ.

To benchmark oral commissure movement tracking, we compared oral commissure movements calculated by FaceADE to those calculated using standard 2D analysis of still frames in ImageJ (Figure 3). Among healthy volunteers, there was concordance between the left and right hemiface in both neutral expression (face in repose) and large smile expressions with dental display (Figure 3A; intraclass correlation coefficient=0.96, 95% CI 0.90-0.98; *P*<.001). The SEs for commissure measurements in the healthy volunteer cohort during a neutral expression on the left and right sides with FaceADE and ImageJ were 0.45, 0.49, 0.72, and 0.70, respectively. The SEs for commissure measurements in the healthy volunteer cohort during a full smile were 0.72, 0.72, 0.77, and 0.62, respectively. Similarly, there was strong alignment between FaceADE and ImageJ in the facial paralysis cohort in both neutral and full smile expressions (Figure 3B). The intraclass correlation coefficient within the facial paralysis cohort showed good reliability at 0.82 (95% CI 0.72-0.88; *P*<.001). The SEs for commissure measurements in the facial paralysis cohort during a neutral expression on the unaffected and affected sides with FaceADE and ImageJ were 0.62, 0.79, 0.85, and 1.00, respectively. The SEs for commissure measurements in the facial paralysis cohort during a full smile were 0.91, 1.06, 1.06, and 1.01, respectively. Bland-Altman analysis (Multimedia Appendix 5) revealed a mean bias of 1.13 mm in the healthy cohort and 0.45 mm in the facial paralysis cohort, indicating that FaceADE measurements were systematically larger than ImageJ measurements. The 95% limits of agreement were −2.13 to 4.38 mm in the healthy cohort and −5.97 to 6.87 mm in the facial paralysis cohort. In total, 97.5% (39/40) of the measurements fell within these limits for the healthy cohort, and 91.2% (73/80) of the measurements fell within these limits for the facial paralysis cohort. Disagreement between FaceADE and ImageJ oral commissure measurement was examined using the Fitzpatrick skin type test (Multimedia Appendix 6), and the median disagreement for all skin types was found to be less than 5 mm.

## Discussion

### Principal Findings

Our findings highlight the feasibility of using IR- and video-based imaging to measure oral commissure symmetry and movement in healthy controls and patients with facial paralysis. In this pilot study, we aimed to benchmark FaceADE’s output against gold-standard 2D image analysis and test the feasibility of using this app during normal clinic workflow.

FaceADE has several advantages compared to existing options. Previously published approaches require a measurement scale to be set using an assumed average value, such as a presupposed iris diameter of 11.77 mm or interpupillary distance of 63 mm. FaceADE’s 3D mesh overlay is individualized to a patient’s face and rapidly created using native iOS software and device hardware [18]. Using both video and IR inputs, FaceADE extracts important temporal data such as the velocity of a facial expression and may be able to quantify difficulty maintaining a facial expression over time. Overall, video-based analysis may provide a more complete assessment of facial movement in comparison to 2D analysis of selected still frames. FaceADE captures the entire facial expression attempt, including maximum movement, average movement, and expression duration. The high intraclass correlation coefficient between FaceADE and gold-standard distance measures is encouraging.

FaceADE is designed to fit into existing clinical workflows. The videos take under 2 minutes to record, and the app is run on the existing iOS platform without the need for additional hardware. The method does not require key frame selection, manual adjustment of key points at anatomical landmarks, or other post hoc modification of images. It also allows for the capture and local storage of videos and still-frame images. With these data, clinicians may be able to use this technology to track patient facial function and recovery over time. A future patient-facing version may allow patients to collect data and share them with their health care team if integrated with a HIPAA-compliant storage platform and electronic medical record.

This feasibility study focused only on oral commissure measurements; therefore, the accuracy of tracking other facial features will need to be validated in future work. Of note, measurements obtained from ImageJ are 2D, whereas FaceADE measures movement in the x-, y-, and z-axes over time. This may at least partially account for the higher distance output by FaceADE than by ImageJ. The ARKit technology that FaceADE leverages is developed by Apple Inc [28]. The accuracy of this method is currently limited to Apple’s proprietary technology and algorithms. While we observed a strong intraclass correlation, the limits of agreement in the Bland-Altman analysis were rather wide, suggesting the need for deeper subgroup analysis to identify weaknesses within the ARKit used in FaceADE. Disease severity or other patient factors may contribute to a higher error rate, and this will be a focus of future study.

Additionally, the sample size in this feasibility study was too small to evaluate accuracy and full clinical utility. Critically, we must assess the reliability and accuracy of FaceADE across patients with different disease severities (as judged by clinician experts using validated metrics such as the Sunnybrook Facial Grading System or eFACE scale) and facial anatomical characteristics, test-retest scenarios, and other common clinical variables. This will be the focus of a future validation study.

While we focused on oral commissure movement for this study as a proof of concept, FaceADE collects data from 1220 vectors on the face. In theory, this permits an exhaustive analysis of facial movement that includes all major facial expressions routinely captured during clinical evaluation of facial paralysis and will provide deeper insights into the diagnosis and treatment of this condition.

### Conclusions

FaceADE offers a feasible and mobile method to use 3D landmarks to track oral commissure movement and symmetry in healthy controls and patients with facial paralysis. This framework could be expanded to provide clinicians with an easy-to-use system to objectively record facial paralysis severity. Video analysis and 3D modeling allow for potentially more accurate and detailed measurements compared to existing technologies.

## Appendix

Multimedia Appendix 1 Video walk-through of the FaceADE app and its features.

Multimedia Appendix 2 Collected FaceADE features.

Multimedia Appendix 3 Standardized prompts used for facial analysis in patients with facial paralysis.

Multimedia Appendix 4 Workflow diagram of the process of data collection, encryption, and analysis.

Multimedia Appendix 5 Bland-Altman analysis of agreement between FaceADE and ImageJ oral commissure measurements.

Multimedia Appendix 6 Distribution of disagreement between FaceADE and ImageJ oral commissure measurements using the Fitzpatrick skin type test.

## Abbreviations

eFACE: Electronic Clinician-Graded Facial Function Scale
HIPAA: Health Insurance Portability and Accountability Act
IR: infrared
REDCap: Research Electronic Data Capture
