# Supplementary material for: Real-Time, Objective Assessment of Facial Paralysis Using a Mobile Tool (FaceADE): Feasibility Case-Control Study
Source: JMIR Form Res. 2026 Jul 14;10:e85965. doi: 10.2196/85965 (PMC13416305; doi:10.2196/85965)
Supplement: Multimedia Appendix 2 [file formative_v10i1e85965_app2.docx]

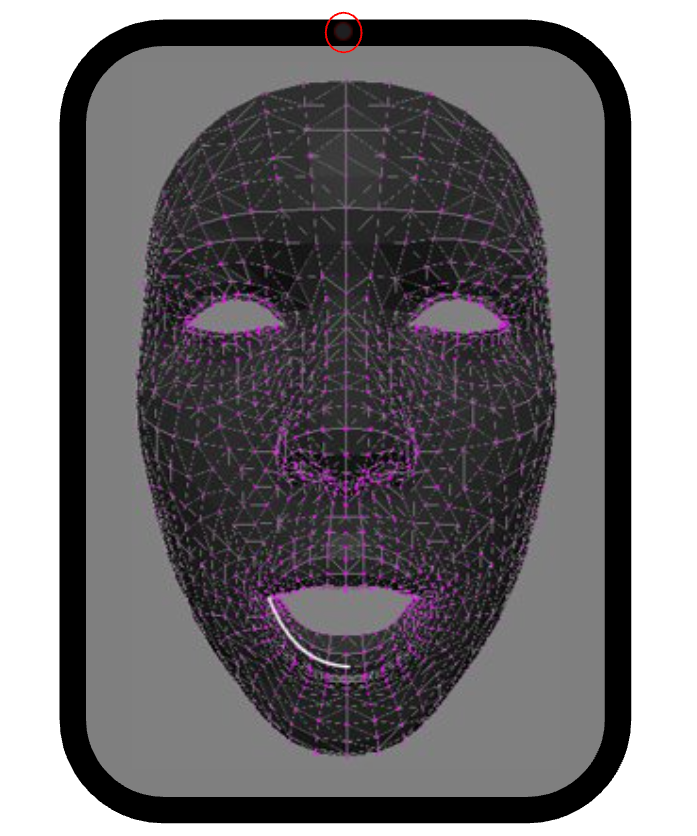


Collected FaceADE features. A projection of all 1220 vectors on the participant’s face by the TrueDepth camera system provided by FaceLandmarks [26]. The white line highlights the feature that is the focus of this study. The lower lip commissure position is the measurement in millimeters from the left or right commissure to the midline of the face along the vermillion border of the lip.
