# Supplementary figures and images for: Real-Time, Objective Assessment of Facial Paralysis Using a Mobile Tool (FaceADE): Feasibility Case-Control Study
Source: JMIR Form Res. 2026 Jul 14;10:e85965. doi: 10.2196/85965 (PMC13416305; doi:10.2196/85965)

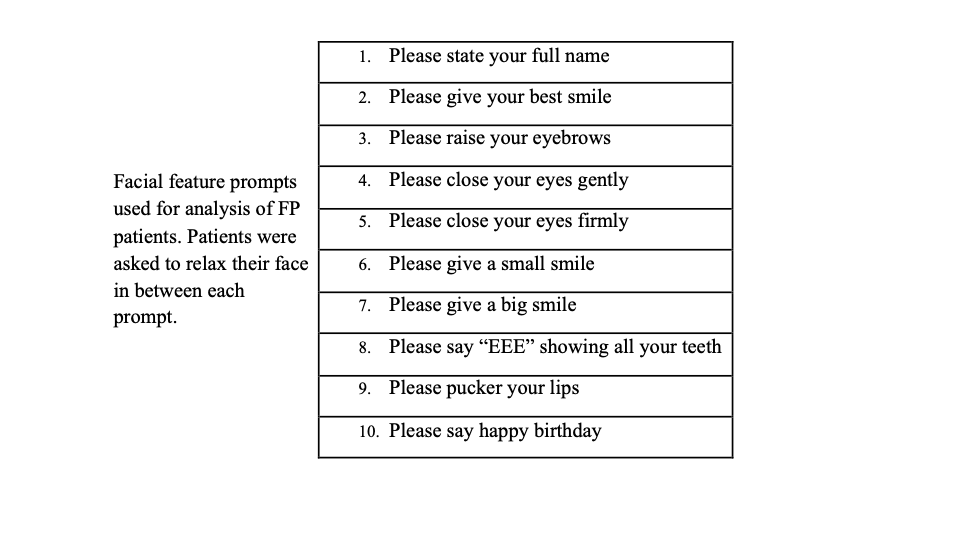

Supplement: Multimedia Appendix 3 [file formative_v10i1e85965_app3.png]

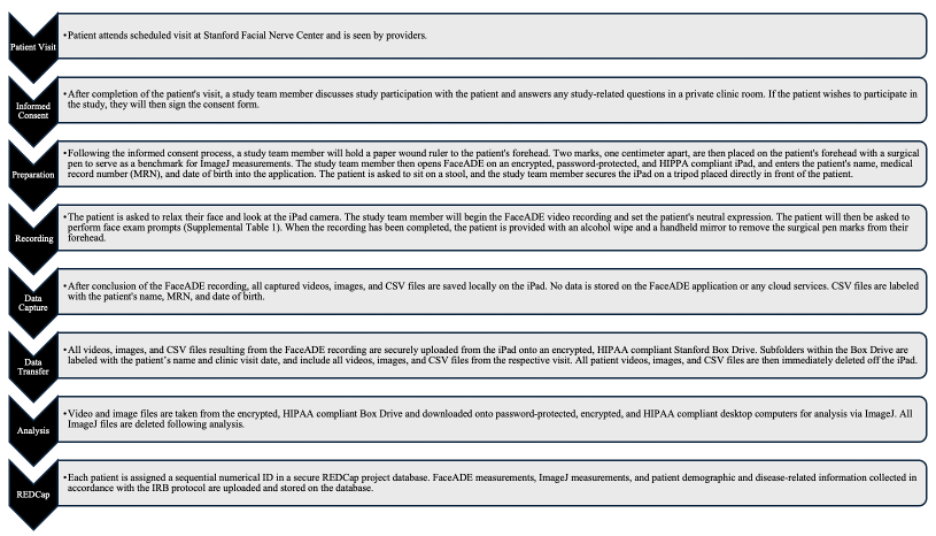

Supplement: Multimedia Appendix 4 [file formative_v10i1e85965_app4.png]
