# Supplementary material for: Real-Time, Objective Assessment of Facial Paralysis Using a Mobile Tool (FaceADE): Feasibility Case-Control Study
Source: JMIR Form Res. 2026 Jul 14;10:e85965. doi: 10.2196/85965 (PMC13416305; doi:10.2196/85965)
Supplement: Multimedia Appendix 5 [file formative_v10i1e85965_app5.docx]

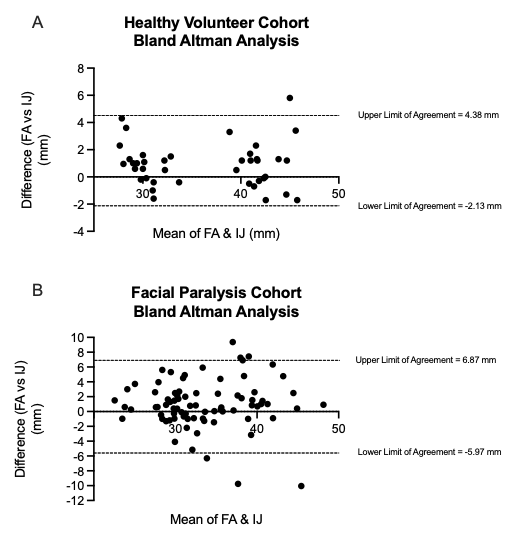


Bland-Altman analysis of agreement between FaceADE and ImageJ oral commissure measurements. The wider limits and lower percentage of agreement in the facial paralysis cohort indicate greater measurement variability between methods in this population.
