# Supplementary material for: Real-Time, Objective Assessment of Facial Paralysis Using a Mobile Tool (FaceADE): Feasibility Case-Control Study
Source: JMIR Form Res. 2026 Jul 14;10:e85965. doi: 10.2196/85965 (PMC13416305; doi:10.2196/85965)
Supplement: Multimedia Appendix 6 [file formative_v10i1e85965_app6.docx]

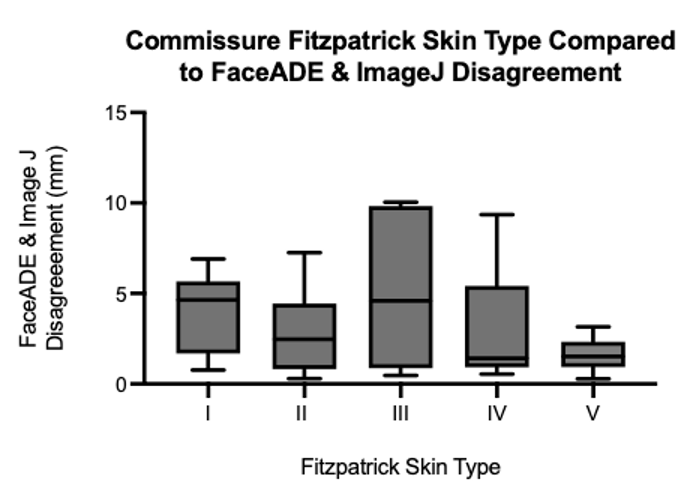


Distribution of disagreement between FaceADE and ImageJ oral commissure measurements using the Fitzpatrick skin type test. The median disagreement in commissure measurements between FaceADE and ImageJ across all Fitzpatrick skin types was less than 5 mm.
